# Supplementary material for: Predictors of loss to follow up from antiretroviral therapy among adolescents with HIV/AIDS in Tanzania
Source: PLoS One. 2022 Jul 20;17(7):e0268825. doi: 10.1371/journal.pone.0268825 (PMC9299289; doi:10.1371/journal.pone.0268825)
Supplement: Attachment — Submitted filename: Review Pone.docx [file pone.0268825.s001.docx]

PLOS One review for “Predictors of loss to follow up from antiretroviral therapy among adolescents with HIV/AIDS in Tanzania

Here the authors describe associated factors with being loss to follow up extrapolated from a national data set over two years for adolescents living with HIV/AIDS.

Strengths: large data set; through and sound analysis,

Areas for improvement:

Major:

Explain in methods where the various settings adolescent can access care (dispensary, health center, hospital) and perhaps a line about what that means to someone not familiar with infrastructure of care delivery in Tanzania)

Discussion includes reviews of other references which support or contradict the findings. But what I want to see is thoughtful ideas or references about why each of the factors such as living in the lake zone or getting care at a dispensary vs a hospital is causing diverse outcomes with loss to follow up. In short I want more in the discussion.

Minor: Grammar and wording in areas needs improvement.
